# Microfluidics-based skin irritation test using *in vitro* 3D angiogenesis platform

**DOI:** 10.1063/1.5093975

**Published:** 2019-07-29

**Authors:** Norhana Jusoh, Jihoon Ko, Noo Li Jeon

**Affiliations:** 1 Department of Mechanical and Aerospace Engineering, Seoul National University, Seoul 08826, South Korea; 2 School of Biomedical Engineering and Health Sciences, Faculty of Engineering, Universiti Teknologi Malaysia, 81310 Johor Bahru, Johor, Malaysia; 3 Institute of Advanced Machines and Design, Seoul National University, Seoul 08826, South Korea; 4 Institute of Bioengineering, Seoul National University, Seoul 08826, South Korea

## Abstract

A global ban on animal experiments has been proposed. Hence, it is imperative to develop alternative models. Artificial skin models should reflect the responses of subcutaneous blood vessels and the immune system to elucidate disease and identify cosmetics' base materials. Notably, *in vivo* skin-irritation cascades involve disruption of the epidermal barrier and the release of proinflammatory mediators in response to chemical stimuli. Such proinflammatory factors promote angiogenesis and blood vessel permeability, as observed in irritant contact dermatitis. As an alternative to animal models, we propose a novel skin-irritation model based on a three-dimensional *in vitro* angiogenesis platform, in which irritated keratinocytes biochemically stimulate vascular endothelial growth factors. Our microfluidic platform hosts interactions between keratinocytes and dermal fibroblasts, which promote angiogenic sprouting. We use sodium lauryl sulfate (SLS) and steartrimonium chloride (SC) as chemical irritants. The irritative effects of SLS and SC are of particular interest due to the ubiquity of both SLS and SC in cosmetics. SLS was observed to significantly affect angiogenic performance, with increasing sprout length. Further promotion of vessel sprouting and lumen formation was observed with 10, 20, and 60 *μ*M of SC, despite its classification as nonirritating and use in supposedly safe formulations. This platform provides an alternative to animal testing as a basis for testing cosmetics and pharmaceutical substances, in addition to serving as a disease model for irritant contact dermatitis.

## I. INTRODUCTION

Skin toxicology testing in the cosmetics and pharmaceutical industries is used to identify whether new compounds are toxic to human skin. One of the qualitative methods used to assess the irritancy potential of chemicals is the hen's egg test-chorioallantoic membrane (HET-CAM) assay, which is based on vascular changes in the chorioallantoic membrane of an egg. Even though the HET-CAM assay can reduce the numbers of animals used, this method cannot completely replace irritation tests in mammals.^1,2^ However, the major challenge in toxicology testing is to define relevant *in vitro* systems, because animal models do not accurately reflect the effects of chemical exposure in humans.^3^ The limitations of animal testing have led to increasing adoption of *in vitro* testing for skin irritation, which is mostly based on the Transwell system.^4,5^

We carried out a validation study of five *in vitro* MTT (3-[4, 5-dimethylthiazol-2-yl]-2, 5 diphenyl tetrazolium bromide) assay developed by the European Center for the Validation of Alternative Methods (ECVAM) and concluded that cytotoxicity measurements alone do not deterministically predict the complexity of skin-irritation cascades.^6^ Exposure to chemicals and cosmetics can lead to a wide variety of skin reactions, including irritant contact dermatitis, which involve inflammatory responses.^7,8^ The inflammatory responses in irritant contact dermatitis are due to skin barrier disruption, cellular changes, and the release of proinflammatory mediators, which are produced mainly by keratinocytes (KCs). These act as initiators in skin inflammatory and immunological reactions, along with Langerhans cells and melanocytes.^9–12^ Furthermore, single or cumulative exposure of the skin to irritants can result in reactions such as edema, inflammation, erythema, dryness, redness, infiltration, scaling, fissuring, and vesiculation.^13^ Therefore, exposure to chemical irritants elevates the secretion of cytokines and growth factors from keratinocytes, including a vascular endothelial growth factor (VEGF), a potent mediator of angiogenesis that stimulates the migration and proliferation of endothelial cells, and facilitates vascular permeability and expression of adhesion molecules in the pathogenesis of irritant contact dermatitis.^8–10,14–16^ Biomarker quantification is necessary for assessing *in vitro* irritant responses due to the absence of visible signs or symptoms compared to *in vivo* testing.^17^ Therefore, biomarker detection methods have been developed for irritant and sensitizer exposure assays in keratinocyte cultures and VEGFs has been suggested as a novel biomarker of keratinocyte damage in skin toxicity testing.^15,17–19^

Along with the growth of microfabrication technologies, the invention of new materials and matrices has enabled the development of organ-on-a-chip models with improved microenvironments and physiological characteristics within microengineered devices.^20^ Advances have been made by numerous researchers in constructing skin-on-a-chip models to reflect the biological complexity of *in vivo* skin.^21–24^ However, these skin-on-a-chip models lack perfusable and functional blood vessels, although they are considered promising tools for drug toxicity studies.^25^

Therefore, the purpose of this paper is to propose a new *in vitro* skin-irritation platform based on angiogenesis responses induced by VEGF upregulation due to damaged keratinocytes, as an alternative to animal models. A new skin-irritation platform was engineered with keratinocytes, dermal fibroblasts, and endothelial cells integrated into a microfluidic device. Designed with multiple channels, the proposed microfluidic device enables coculturing of endothelial cells and keratinocytes with two incompatible media. As a proof of concept, keratinocytes were treated with sodium lauryl sulfate (SLS), a well-known irritant, so that we could mimic the skin-irritation mechanism in a microfluidic chip. Human skin cells, either in a monolayer culture or an organ culture, produce various types of irritation such as redness, dryness, edema, and scaly crusts when exposed to certain chemical agents, including SLS.^13^ As a surfactant molecule that elicits an irritant effect, SLS can penetrate the epidermis and be incorporated into the stratum corneum lipids. This results in irritant contact dermatitis.^26^ SLS affects the integrity of the skin barrier and consequently induces inflammatory cytokines, which are produced by keratinocytes.^9,27^

In addition, we used steartrimonium chloride (SC), which is considered nonirritating and used in safe formulations to demonstrate the potential of our microfluidic platform for studying the irritation mechanisms of agents that have not been clinically reported to induce skin irritation. Numerous chemicals are used as cosmetic ingredients, but few have been studied intensively in the context of cosmetics testing because these ingredients were determined to be safe when formulated, and their concentrations were thought to be nonirritating.^28^ This situation resulted in a lack of clinical reports on irritation by or sensitization to several types of chemical ingredients, including the trimonium compounds, which are in hundreds of products.^28^ Steartrimonium chloride is one of three quaternary ammonium salts used as cosmetic ingredients in surfactant-cleansing, hair-conditioning, and antistatic products.^28^ To the best of our knowledge, there have been no reports on the use of this chemical in *in vitro* skin-irritation testing; thus, the irritation mechanism of this type of chemical has not been well studied.

## II. RESULTS

### A. Microfluidic device configuration

**FIG. 1.**
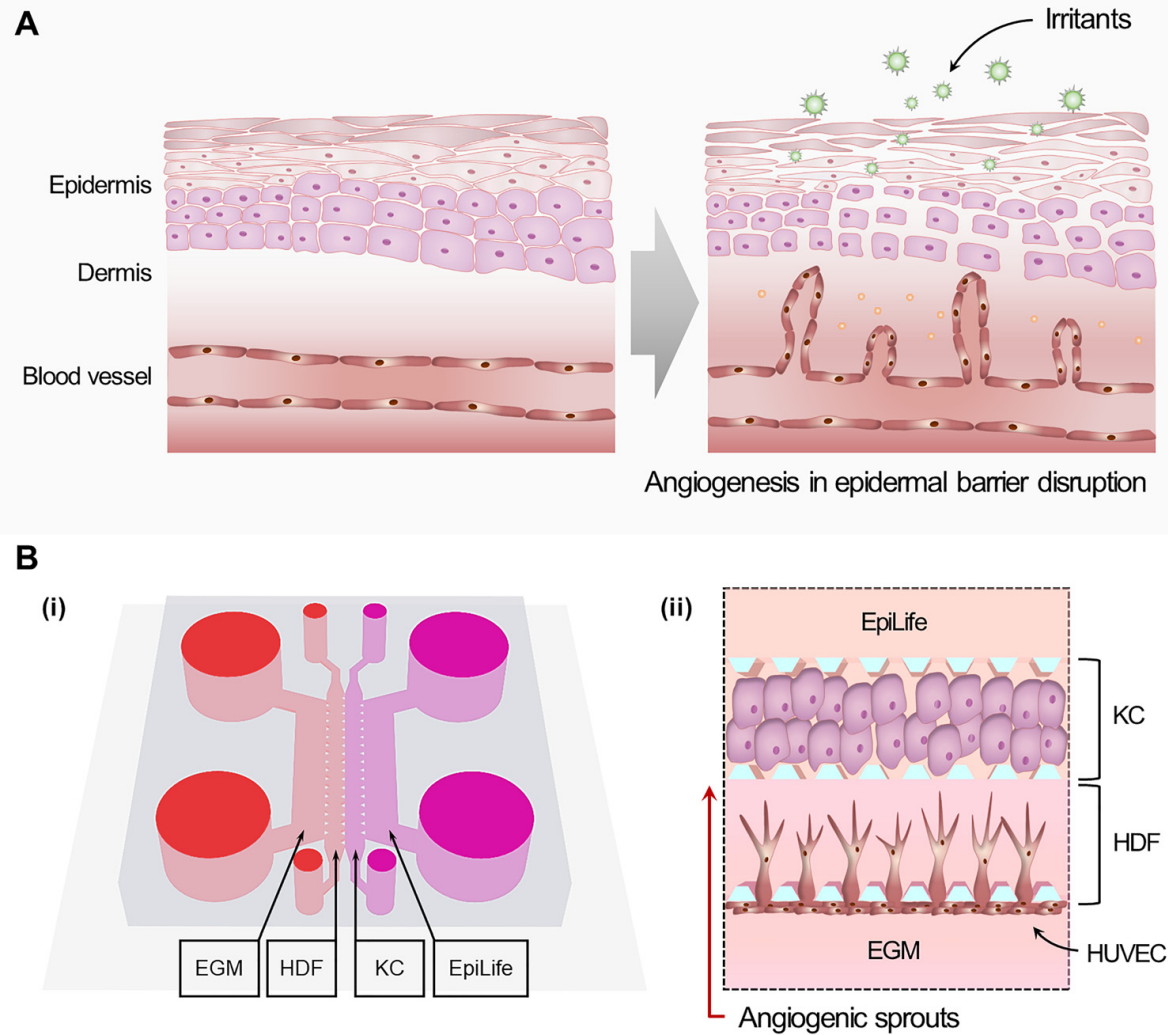
Skin microenvironment incorporating a microvascular system for skin-irritation testing using a microfluidic device. (a) Disruption of the epidermal barrier resulting in release of chemokines and cytokines in irritant contact dermatitis. (b) Schematic of the device and cell configuration for the proposed skin irritation test model based on angiogenesis responses in a microfluidic platform. (i) An illustration showing the composition of HDF, KC, and each specific medium in the microchannel. (ii) Angiogenic responses occur toward the KC layer as a space where the HDF is cultured.

The angiogenesis mechanism response to chemical irritants can be explained in terms of the upregulation of VEGFs due to the disruption of the epidermal barrier, as shown in Fig. 1(a). Under quiescent conditions, keratinocytes secrete growth factors via autocrine and paracrine signaling to maintain cell-cell interactions and various physiological functions. Consequently, exposure to chemical irritants will elevate cytokine and growth factor secretion from keratinocytes due to cellular damage. Skin irritation can be evaluated by observing elevations in blood vessel sprouting, which are related to the release of cytokines and growth factors during the inflammation process. Figure 1(b) shows the new platform for evaluating skin irritation based on angiogenic responses. The microfluidic platform consists of multiple channels that mimic the structure of the dermal and epidermal layers, with endothelial cells representing the blood vessels of the human skin. As shown in Fig. 1(b), the microfluidic device consists of two 800-*μ*m-wide channels separated by 100-*μ*m microposts to capture the hydrogels while simultaneously enabling the diffusion of the medium from the side channels. Using this platform, we positioned keratinocytes to stimulate the sprouting of HUVEC from the wall to the left of the HDF-fibrin matrix via secreted growth factors. Furthermore, we used side-by-side channels of HDFs and human keratinocytes to facilitate the cell-cell interactions that induce angiogenesis from endothelial cells attached to the boundary of the fibrin bed. Consequently, we observed enhanced vessel sprouting when keratinocytes were exposed to chemical irritants.

### B. Effect of quiescent keratinocytes on angiogenesis

**FIG. 2.**
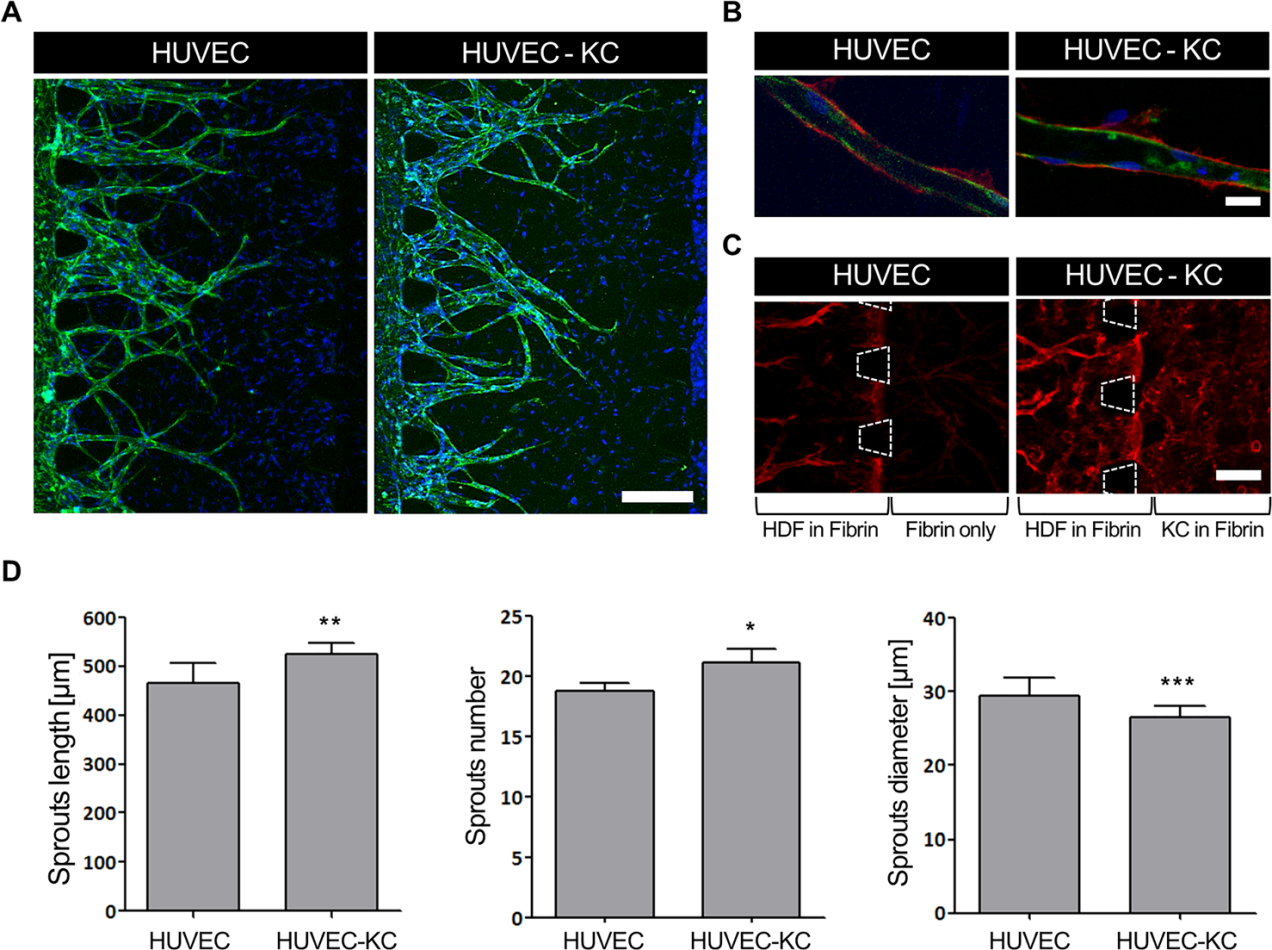
Angiogenesis formation at quiescence with HDFs and KCs side by side. (a) Representative images of angiogenesis sprouting. The KC channel was filled with an acellular fibrin matrix as a control (HUVEC), and with keratinocytes in the fibrin matrix for HUVEC-KCs. Scale bar = 100 *μ*m. (b) Lumen formation enclosed by endothelial cells. Scale bar = 20 *μ*m. (c) Collagen-IV deposition near microposts between HDF and KC channels. Scale bar = 100 *μ*m. (d) Quantification of the vessel sprout length, number, and diameter (n = 8). All samples were stained with CD31 (green), nuclei (blue), and collagen IV (red).

While chemical stresses can upregulate the secretion of various cytokines and growth factors, keratinocytes also produce some cytokines constitutively under quiescent conditions.^8–10,14,16^ Figure S3 in the supplementary material shows the effect of incorporating the HDF in inducing the vessel formation. Without keratinocytes at the side channel, the sprout length and sprout number did not have significant differences between the fibrin and fibrin-HDF platform. However, with keratinocytes at the side channel, there was a significant increase in the sprout length for the fibrin-HDF platform as compared to the fibrin platform. Therefore, we initially observed the effects of HDF and keratinocyte communication on the enhancement of angiogenesis under sprouting quiescent conditions, as shown in Figs. 2(a)–2(c). Generally, perfusable blood vessels with lumen formation were observed using CD31 and collagen IV markers under both conditions, as shown in Figs. 2(a) and 2(b). We investigated the collagen-producing effect of keratinocytes at the microposts that separated the HDFs and keratinocytes, as shown in Fig. 2(c). Denser collagen IV deposition was clearly observed under the keratinocyte interaction conditions than without keratinocytes. For a detailed comparison, Fig. 2(d) shows the lengths, numbers, and diameters of the vessels sprouting within the fibrin-HDF matrix under quiescent conditions, both with and without keratinocytes. With keratinocytes, the average sprout length increased from 466 ± 16 to 524 ± 10 *μ*m and the average number of sprouts increased significantly, from 19 ± 1 to 21 ± 1 *μ*m. However, the average diameters of the vessels decreased from 30 ± 1 to 27 ± 1 *μ*m with keratinocyte interactions. In conclusion, quiescent keratinocytes enhanced the lengths and numbers of sprouting vessels, but not their diameters.

### C. Effect of SLS irritation on keratinocyte viability

**FIG. 3.**
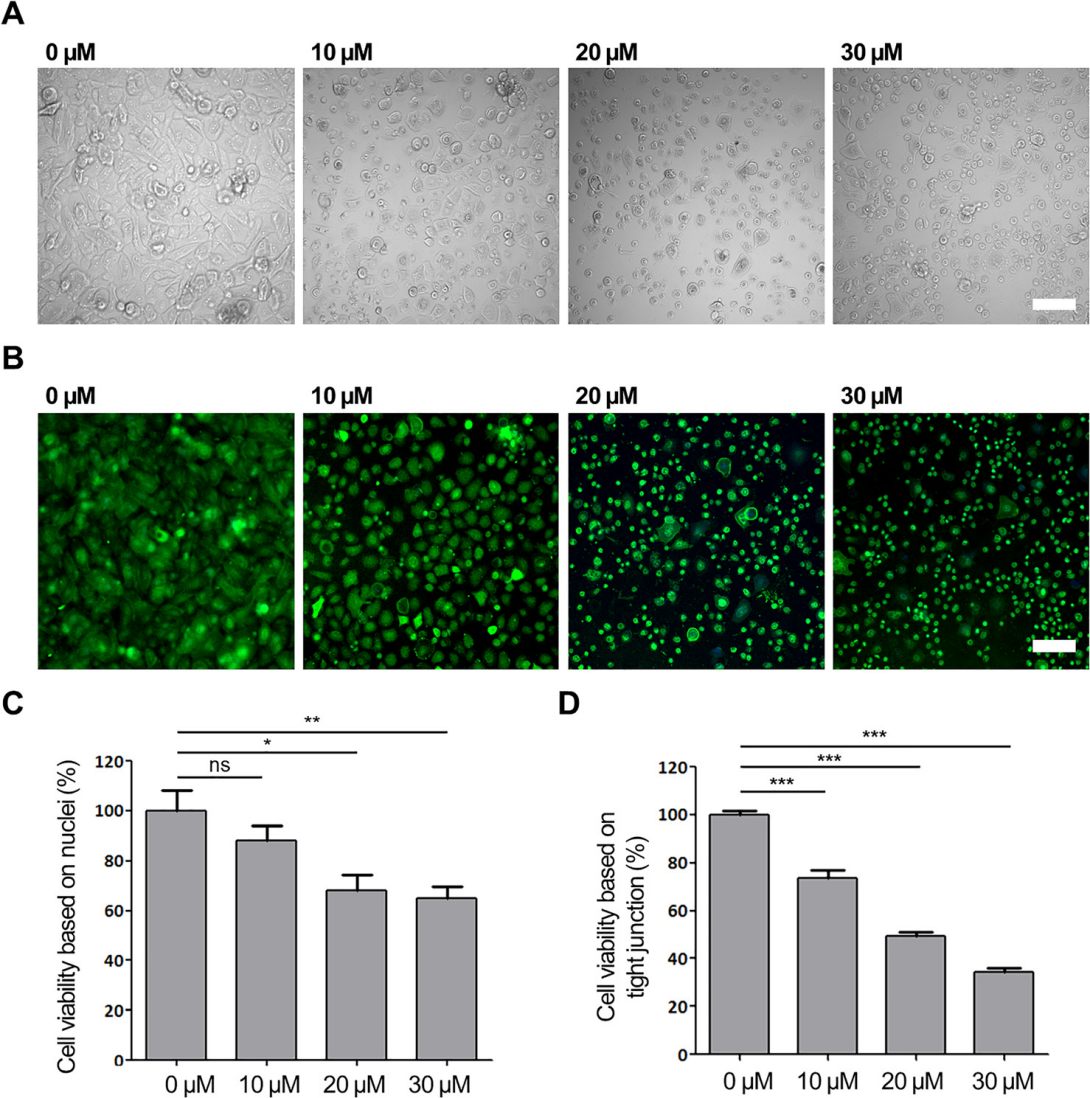
Verification of keratinocyte viability by 4-day sodium lauryl sulfate (SLS) treatment in a 24-well plate. (a) and (b) Representative bright field and confocal microscopic images of keratinocytes by concentration. Cells were stained with Claudin-1 (green) and nuclei (blue). Scale bar = 100 *μ*m. (c) and (d) Quantitative analysis of nuclei and tight junctions of viable keratinocytes (n = 4).

**FIG. 4.**
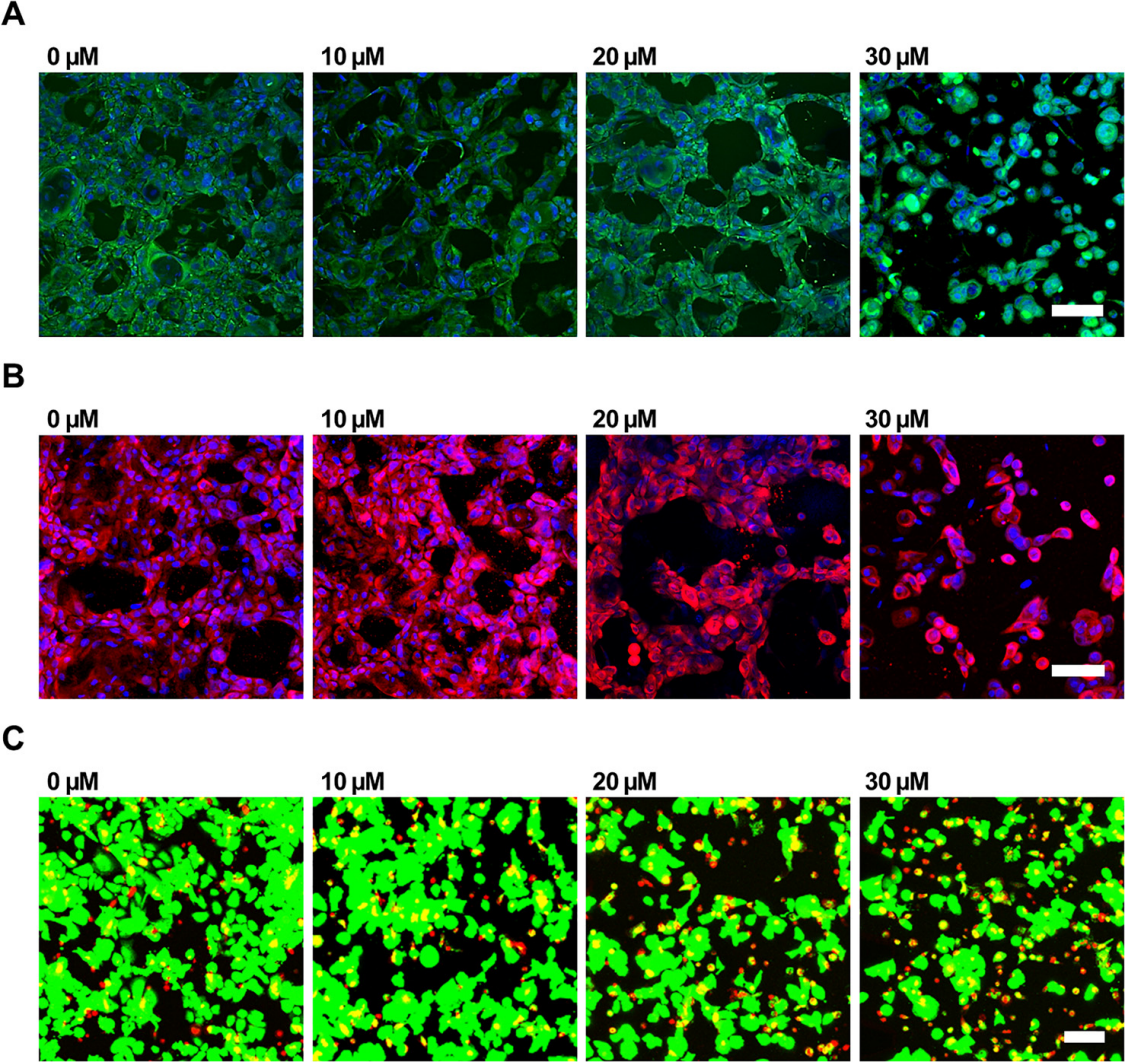
Effect of SLS on keratinocytes after 4 days of exposure in the microfluidic platform. Representative images of keratinocytes stained with (a) Claudin-1 (green) for tight junction, (b) keratin 14 (red) for keratin, and (c) live/dead assay with calcein-AM (green) for live cells and ethidium-1 (EthD-1) (red) for dead cells. Scale bar = 100 *μ*m.

Firstly, we studied the effect of SLS on keratinocyte viability in both a 2D monolayer and 3D fibrin. We observed the 2D keratinocyte monolayer after 4 days of SLS exposure in 24 wells, as shown in the bright-field images [Fig. 3(a)] and confocal images [Fig. 3(b)], alongside a quantification of the cell viability [Figs. 3(c) and 3(d)]. The SLS treatment disturbed the keratinocyte cell proliferation, with cells becoming detached from each other and undergoing cell death, as shown by the gradual decrease in Claudin-1 expression. Figures 3(c) and 3(d) show the cell viability, quantified based on the areas of the nuclei and tight junctions after treatment. Compared to the control samples, the area of the nuclei decreased significantly, by 88%, 68%, and 65% after treatment with 10, 20, and 30 *μ*M of SLS, respectively. At the same time, the areas of the tight junctions decreased significantly, by 74%, 50%, and 35% after treatment with 10, 20, and 30 *μ*M of SLS, respectively. Figure 4 shows confocal images of keratinocytes in the 3D fibrin hydrogel after 4 days of SLS treatment.

Generally, keratinocyte proliferation decreased as the SLS concentration increased. We inferred this from Claudin-1, K14 and live/dead assays, as shown in Fig. 4. In the case of 3D keratinocytes, the expression of Claudin-1, representing tight junctions, and K14, indicating keratin, decreased with increasing SLS concentration. The live/dead assay showed that the numbers of dead cells increased with the SLS concentration by observing the cell shape. The cell death was gradually started at 10 and 20 *μ*M of SLS. Most of the keratinocytes changed shape, resulting in cell death at 30 *μ*M of SLS. SLS can damage the structure of proteins such as keratin, involucrin, profilaggrin, and loricrin, exposing new water-binding sites and causing hyperhydration of the stratum corneum and disorganization of the lipid bilayers.^9^ It has been reported that cumulative application of SLS resulted in downregulation of claudin gene expression in humans.^31^ These findings were supported by a previous report that SLS affects the survival rate of keratinocytes.^13,14^

### D. Effect of SLS irritation on angiogenesis

**FIG. 5.**
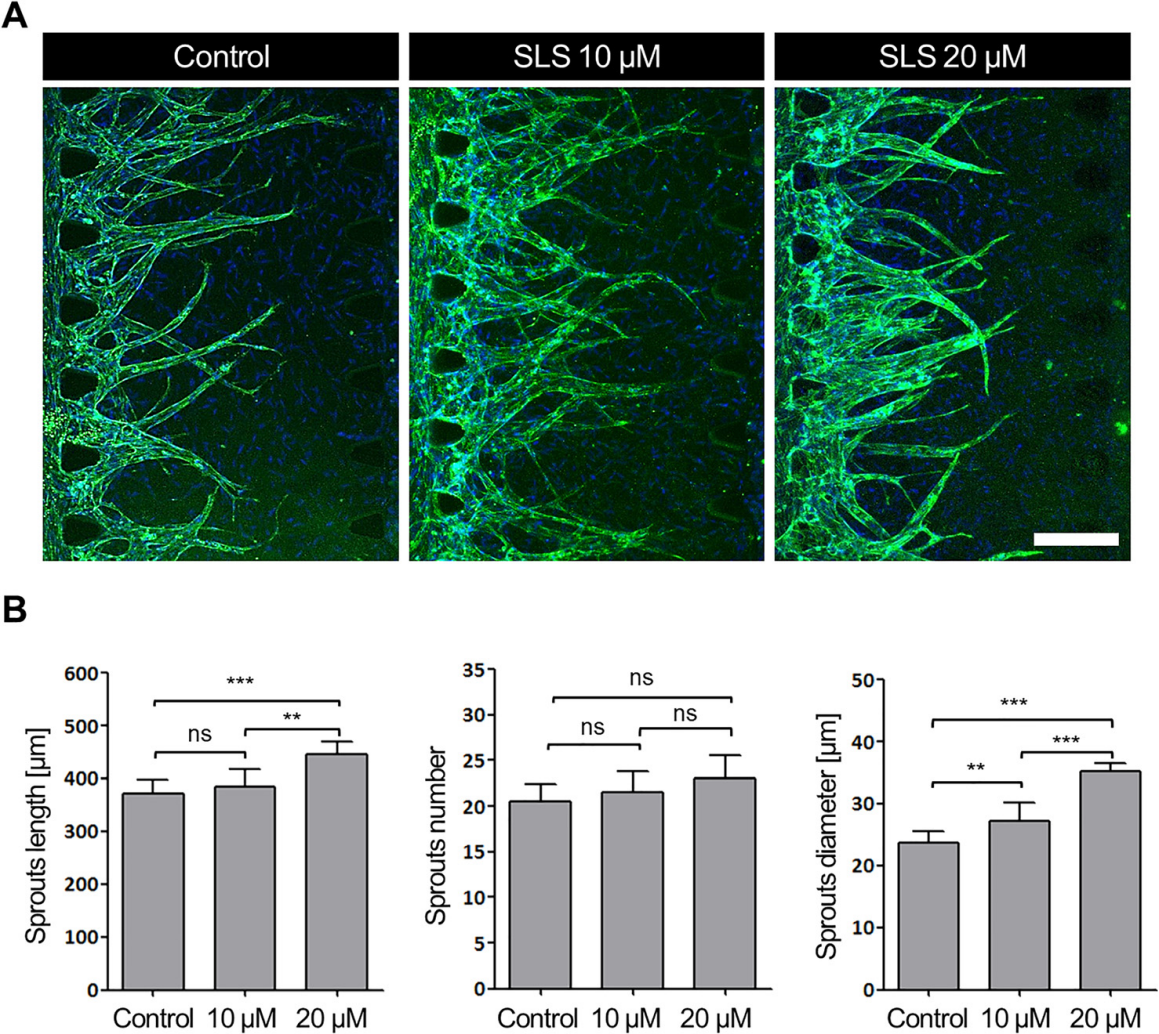
Investigation of angiogenic effects at different SLS concentrations after 4 days of exposure in the microfluidic platform. (a) Representative confocal microscopy images showing the angiogenic response. Samples were stained with CD31 (green) and nuclei (blue). Scale bar = 200 *μ*m. (b) Quantification of angiogenesis sprouting with microvessel length, number, and diameter as criteria (n = 4).

Even though cell viability is a common indicator of the toxicity of chemicals, measurement of pathogenic biomarkers such as VEGFs and other cytokines from keratinocytes may improve our ability to determine the toxic potential of chemicals.^13,15,32^ On the other hand, the VEGF is the main agent responsible for angiogenesis and vascular permeability. Instead of measuring cytokine levels, we observed angiogenesis cascades in skin irritation directly. We used a microfluidic device as a skin-toxicity platform to investigate the effect of SLS irritants on VEGFs via angiogenesis formation, as shown in Fig. 5.

As shown in Fig. 4, most of the keratinocytes in fibrin were dead after exposure to 30 *μ*M SLS. Therefore, we applied SLS concentrations only between 0 and 20 *μ*M when evaluating the effect of chemical irritants on angiogenesis in our microfluidic platform. Figure 5(a) shows confocal images of angiogenesis and Fig. 5(b) shows the average length, number, and diameter of sprouts after 4 days of SLS treatment. Compared to 10 *μ*M, 20 *μ*M had a more significant effect on the length of sprouts with respect to the control condition, 0 *μ*M. The average sprout length increased from 372 ± 12 to 446 ± 14 *μ*m after 20-*μ*M SLS treatment, and reached 384 ± 1 *μ*m after 10-*μ*M SLS treatment. However, the numbers of angiogenesis sprouts were relatively similar under all conditions, being 21 ± 1, 22 ± 1, and 23 ± 1 for 0, 10, and 20 *μ*M SLS, respectively. Although the numbers of sprouts did not vary significantly, the diameters of the sprouts varied significantly among these three conditions. The average diameters of the sprouts were 24 ± 1, 27 ± 1, and 35 ± 1 *μ*m for 0, 10, and 20 *μ*M of SLS, respectively. Based on the observed data, the effect of SLS on angiogenesis morphogenesis indicated its skin-irritation mechanism.

### E. Angiogenesis mechanism with SC

**FIG. 6.**
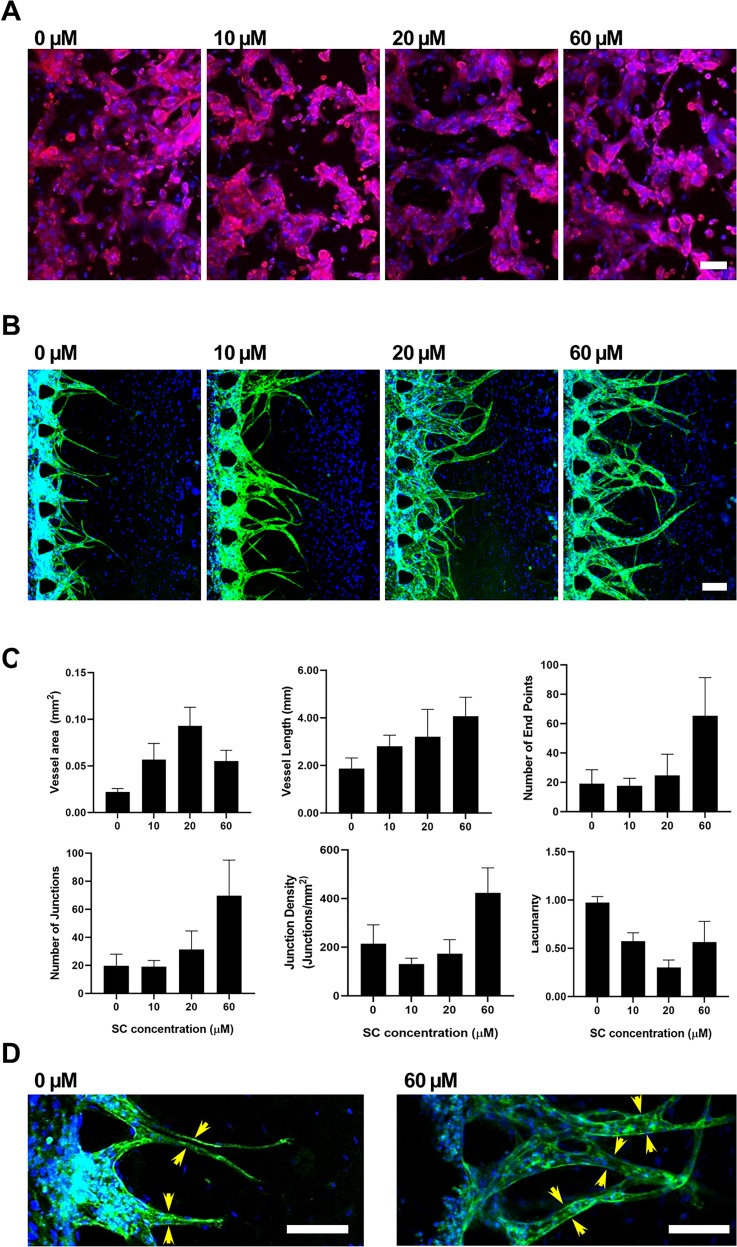
Investigations of angiogenic effects at different SC concentrations after 4 days of exposure in the microfluidic platform. (a) Representative images of keratinocytes stained with keratin 14 (red) for keratin. (b) Representative images showing the angiogenic response. Samples were stained with CD31 (green), and nuclei (blue). Scale bar = 100 *μ*m. (c) Quantification of vascular sprouting by using Angiotool to quantify vessel area, vessel length, number of end points, number of junctions, junction density, and lacunarity (n = 3). (d) Representative images of lumen formation (yellow arrow) after 2 days of treatment. Scale bar = 100 *μ*m.

Next, we investigated the effect of SC, an uncommon irritant, on the angiogenesis response, as shown in Fig. 6. We observed the effects of SC on keratinocyte proliferation and angiogenesis. As shown in Fig. 6(a), an increase in SC concentration affected keratinocyte death, as observed by the reduction in the K14 marker. After exposure to 100 *μ*M of SC, most keratinocytes died, as shown by the changes in the cell shape. At 10, 20, and 60 *μ*M of SC, keratinocyte morphology did not show much difference, but angiogenesis was completely different. This demonstrates the advantage of evaluating angiogenesis responses for carrying out irritation assays. Briefly, Fig. 6(b) shows the enhanced vessel sprouting and Fig. 6(c) shows the quantification of angiogenesis vessels based on Angiotool (Fig. S4 in the supplementary material) to quantify the vessel area, vessel length, number of end points, number of junctions, junction density, and lacunarity (a metric of vessel nonuniformity). Figure 6(d) clearly shows the representative lumen formation for 0 *μ*M of SC and 60 *μ*M of SC concentration. The average vessel area was increased from 0.02 mm up to 0.09 mm for 20 *μ*M of SC, but it was decreased to 0.06 mm at 60 *μ*M of SC. However, the vessel length was increased steadily from 1.87 mm at 0 *μ*M of SC to 4.07 mm at 60 *μ*M of SC. Nevertheless, the number of end points and number of junctions show rapid increment at 60 *μ*M of SC compared to the other conditions. At 60 *μ*M of SC, the number of junctions was 70 compared to 31 at 20 *μ*M of SC. The number of endpoints was increased from 25 at 0 *μ*M of SC to 65 at 60 *μ*M of SC. Meanwhile, junction density and lacunarity did not show any specific trends with the increase of SC concentration. Junction density was decreased from 215.48 junction/mm^2^ at 0 *μ*M of SC to 130.93 junction/mm^2^ at 10 *μ*M of SC. However, the density was increased again to 173.81 junction/mm^2^ at 20 *μ*M of SC and 423 junction/mm^2^ at 60 *μ*M of SC. For lacunarity, the highest lacunarity was 0.97 at 0 *μ*M of SC while the lowest was 0.30 at 20 *μ*M of SC.

This preliminary finding is supported by previous animal studies, which demonstrated that SC induces skin irritation. In early assessments, SC yielded positive results in an *in vivo* skin-irritation study using guinea pigs.^33^ Later, another assessment was carried out based on a single application of SC to albino rabbits, which showed that 79.2% SC was irritating after 1 h of exposure, with erythema persisting for up to 22 days and edema for up to 7 days.^34^

## III. DISCUSSION

In this paper, we propose a new skin-irritation model based on the responses of angiogenesis in a microfluidic platform as an alternative *in vitro* irritant-testing device. This model incorporates dermal fibroblasts, endothelial cells, and keratinocytes in designated microfluidic channels, which maintain autocrine and paracrine communication. In addition to enabling cell separation, multiple channels allow different media within a single device. This is crucial for maintaining the viability of multiple cell types. The microfluidic device is tuned simultaneously to biochemical stimulation, resulting in discrete changes in growth factor concentrations and gradients. These are important during the sprout-elongation and lumen-formation stages.^35^ The formation of lumens is important for transporting nutrients, oxygen, and waste products to and from tissues, and the diameter of the lumens increases from 5 *μ*m to 10 *μ*m in humans.^36^ For angiogenesis, once sprouting has been initiated, the tip cells guide the growth of sprouts across the fibrin matrix until they reach the opposite end of the channel. Further development of the vasculature results in interconnected networks that occupy extended areas of the fibrin matrix, followed by the enlargement of lumina. The process of new blood vessel formation requires the establishment of a functional vascular loop to promote the survival and stabilization of newly formed vessels, as well as physiological function and homeostasis.^29^

Compared to other microfluidic devices used to study blood vessels, our platform enables us to seed dermal fibroblasts and keratinocytes side by side in the microchannels, which mimic the *in vivo* structure of skin. In this study, keratinocytes were cultured in a three-dimensional (3D) fibrin gel, whereas keratinocytes are usually cultured under two-dimensional (2D) conditions to reconstruct the epidermis. Fibrin is a fibrous, nonglobular protein that is closely associated with fibronectin in blood clotting; fibronectin is a glycoprotein found in the epidermal extracellular matrix.^37^ Keratinocytes in fibrin have been used in skin reconstruction as epidermis, for coverage of massive burns and as an epitheliallike layer.^38^ Fibrin has been demonstrated to be a suitable substrate for keratinocyte cultivation and transplantation and has the ability to maintain stem cell populations after *in vivo* transplantation of keratinocytes.^39^

Our results showed that blood-vessel formation was enhanced by coculturing dermal fibroblasts with quiescent keratinocytes. It was reported previously that growth factors secreted from fibroblasts in a side channel diffuse along a gradient, thus stimulating angiogenesis in microfluidic devices. Similar to fibroblasts, various types of VEGFs at low to moderate expression levels have been observed extracellularly in cultured keratinocytes.^40–42^ Therefore, as an endothelial and microvessel hyperpermeability growth factor, the VEGF secreted by keratinocytes may stimulate vessel formation in angiogenesis due to paracrine communication between endothelial cells and keratinocytes under quiescent conditions. Furthermore, mesenchymal-epithelial communication between human keratinocytes and human fibroblasts regulates growth factor secretion, which may stimulate blood-vessel formation under quiescent conditions.

Epidermal keratinocytes induce fibroblast activities, thus producing soluble factors and hormones to enhance keratinocyte proliferation and migration.^40–42^ This mesenchymal-epithelial paracrine interaction has been reported to regulate angiogenesis in skin homeostasis, wound healing, inflammation, and tumor promotion.^43–45^ The *in vivo* dermis-epidermis junction is composed of basal lamina proteins such as collagen IV, laminin, and bullous pemphigoid antigen (BPA).^46,47^ Keratinocytes have the ability to produce numerous collagenase-inducing cytokines,^45^ and also secrete most of the components of the basal lamina, including laminin, collagen IV, and collagen VII.^46,47^ In addition to stimulating basal-membrane components directly, dermal fibroblasts also affect the laminin, collagen IV and collagen VII expression of keratinocytes via keratinocyte–fibroblast interactions.^46,48,49^ Therefore, in our device, keratinocyte and dermal fibroblast interactions contribute to the expression of collagen at the boundaries of the microposts.

We also successfully demonstrated the enhancement of angiogenesis vessels due to keratinocytes during exposure to irritants. This mimics the eczematous reaction that occurs in irritant contact dermatitis. Single or cumulative exposure to chemical irritants leads to irritant contact dermatitis, which involves skin-barrier disruption, cellular changes, and the release of proinflammatory mediators.^9,27^ Thus, disordered keratinocyte proliferation due to skin inflammation and altered tight-junction protein expression leads to stratum corneum barrier damage with epidermal-barrier function disruption and irritation, both of which play significant roles in skin-barrier damage.^9,27,50^ In addition to suppressing growth, contact irritants are cytotoxic to keratinocytes and induce histological changes such as hyperplasia, incomplete keratinization, loss of the granular layer, acantholysis, and necrosis in organ-cultured skin, even at low concentrations.^13^ The observed eczematous reaction involved cellular changes and the release of proinflammatory mediators, thus implicating the VEGF in the pathogenesis of irritant contact dermatitis.^9,50–52^ This induction of the VEGF occurred via the activation of epidermal growth factor receptor (EGFR) and metalloprotease activity, which took place after the keratinocytes were exposed to the chemical irritant.^53^ Compared to unexposed skin, this molecular event enhanced VEGF levels after either single or repeated exposure.^52,54^

Specifically, SLS significantly increased the VEGF in keratinocytes by affecting the gene transcription of the angiogenic VEGF, which is the main growth factor for vessel formation during angiogenesis.^15^ It was reported that *in vitro* VEGF levels increased almost fivefold after a single exposure of SLS to human keratinocytes.^54^

The VEGF is a potent mediator of angiogenesis, which stimulates the migration and proliferation of endothelial cells, facilitates vascular permeability, and induces expression of adhesion molecules by endothelial cells.^51^ Therefore, the increase in the level of the VEGF during skin irritation promotes angiogenesis. We observed that both SLS and SC influenced angiogenic morphogenesis and can thus be used as representative models of the skin-irritation mechanism.

## IV. CONCLUSION

Our microfluidic platform has the potential to demonstrate the complex angiogenesis mechanisms induced by irritant contact dermatitis pathophysiology. Autocrine and paracrine communication between keratinocytes and dermal fibroblasts enhanced angiogenesis vessel sprouting and increased collagen deposition under quiescent conditions. This new platform for investigating skin irritation is based on the enhancement of angiogenesis due to the effects of chemical irritants on keratinocytes. SLS and SC behaved similarly with respect to angiogenesis formation. This implies that our platform provides a novel approach that could potentially replace current avascular skin-irritation models. Thus, these findings demonstrate that our platform is applicable to studying the mechanisms of both common and uncommon irritants. Hence, it can be used to support existing *in vitro* and clinical skin-irritation tests. In addition to irritants, this platform can be used to test other types of biochemical stimuli, such as allergens or corrosives. As such, it could potentially be applied as an assay for safety evaluation of cosmetics- and drug-testing applications.

## V. METHODS

### A. Microfluidic device fabrication

Microfluidic chips were fabricated using polydimethylsiloxane (PDMS, Sylgard 184; Dow Corning) with channel structures patterned by standard photolithography and soft lithography. The microfluidic design was based on a single-channel perfusable-blood-vessel device.^29^ In this study, the device was modified to have double channels, to allow direct contact between human dermal fibroblasts (HDF) and keratinocytes. Figure S1 in the supplementary material shows the modification of the single channel device to double channel device. Compared to the single channel device, the double channel has a direct interface between the HDF channel and keratinocyte channel, as shown in Figs. S1 and S2. As shown in Fig. 1(b), the device was designed to mimic skin angiogenesis based on irritant stimulation by coculturing human umbilical vein endothelial cells (HUVEC), HDF, and keratinocytes in a microfluidic device. There was no requirement for ethics approval for the use of these human cells.

### B. Cell culture and cell seeding

Human epidermal keratinocytes derived from neonatal skin were purchased Gibco. Human keratinocytes were cultured in Epilife supported with keratinocyte growth supplement (HKGS) and passage 3 was used for the experiment. Human umbilical vein endothelial cells (HUVEC) derived from human umbilical vein were purchased from Lonza. HUVEC were cultured in endothelial growth medium (EGM) supported with an EGM-2 bullet kit and passage 4 was used for the experiment. Human dermal fibroblasts derived from human skin were purchased from Lonza. HDF were cultured in fibroblast growth medium (FGM) supported with FGM-2 bullet kit and passage 6 was used for the experiment. All cells were incubated at 37 °C in a humidified atmosphere of 5% CO_2_. The medium was refreshed every 2 days.

Angiogenesis and cell seeding were conducted based on an established procedure,^29^ as shown in Fig. 1(b). Generally, the fibrin matrix was prepared by mixing 2.5 mg/ml bovine fibrinogen (Sigma) and aprotinin (0.15 U/ml; Sigma) prior to mixing with the cell suspension. The final mixture was supplemented with thrombin (0.5 U/ml; Sigma) and left to clot at room temperature for 5 min. The left channel was filled with the fibrin-HDF matrix by mixing fibrin with 2 × 10^6^ cells/ml HDF in endothelial growth medium (EGM). Dissociated human keratinocytes were suspended in EpiLife medium at 5 × 10^6^ cells/ml before mixing with the fibrin matrix, and were then injected into the right channel. After fibrin gel polymerization, EGM and EpiLife medium were added to the designated reservoirs. The device was incubated for 24 h (37 °C, 5% CO_2_), to dissipate air bubbles and allow fibroblasts to become established within the fibrin gel matrix. On the following day, HUVEC suspended in EGM at a concentration of 5 × 10^6^ cells/ml were adhered to the fibrin-HDF surface of the left channel by tilting the microfluidic chip to 90° for 30 min.

### C. Effect of SLS on keratinocyte viability

After 2 days of cell seeding in the microfluidic channel, keratinocytes (5 × 10^6^ cells/ml) in fibrin were treated with 0, 10, 20, or 30 *μ*M SLS by adding SLS (Micolin S490; Miwon Commercial Co., Ltd.,) stock solution into the right EpiLife medium reservoir. Medium in the left reservoir was maintained with EGM. Samples were observed for cell-viability properties such as the tight junction, basal keratin, and live/dead assays. For cell-viability quantification after SLS treatment, keratinocytes (0.05 × 10^6^ cells/ml) were cultured in 24-well plates for a few days until confluence was reached, and then treated with a final solution of 0, 10, 20, or 30 *μ*M SLS in EpiLife for 4 days.

### D. Effect of SLS on angiogenesis

After HUVEC seeding overnight, keratinocytes in fibrin were treated with 0, 10, or 20 *μ*M SLS by adding SLS stock solution into the right EpiLife medium reservoir, as shown in Fig. 1(b). Medium in the left reservoir was maintained with EGM. SLS treatment was conducted for 4 days; regular medium was changed after 2 days. Samples were fixed and stained for further analysis of vessel sprouting.

### E. Effect of SC on angiogenesis

After 2 days of cell seeding in the microfluidic channel, keratinocytes (5 × 10^6^ cells/ml) in fibrin were treated with 0, 10, 20, or 60 *μ*M SC to observe the effect on keratinocyte proliferation. To observe effects on angiogenesis formation, after HUVEC were seeded overnight, keratinocytes (5 × 10^6^ cells/ml) in fibrin were treated with 0, 10, 20, or 60 *μ*M of SC [Miconium STAC 80(I)] by adding SC into the right EpiLife medium reservoir. Medium in the left reservoir was maintained with EGM.

### F. Immunostaining and imaging

For blood vessel imaging, fixed samples were stained and incubated overnight at 4 °C with mouse monoclonal antibodies specific for CD31 [Alexa Fluor 488 was purchased from BioLegend (conjugated with a fluorescent marker)] at a dilution of 1:200. On the following day, samples were subjected to nucleus staining with Hoechst 33342 (1:1000) for 1 h of incubation at room temperature. For basal keratin imaging, samples were stained with Keratin 14 (K14) primary antibody (1:500) overnight, followed by a second antibody (1:500) and Hoechst 33342 for 4 h at 4 °C. For collagen IV imaging, samples were stained with collagen IV unconjugated antibody followed by incubation with fluorescence-conjugated secondary antibodies (1:1000) for 4 h at 4 °C. For tight-junction imaging, fixed samples were stained with Claudin-1 (1:200) overnight, followed by Hoechst 33342 for further qualitative and quantitative analyses. All samples were washed three times and stored in phosphate-buffered saline (PBS) before imaging. For live/dead assay imaging, live samples were stained directly using Live Dead Assay Kits (Invitrogen/Molecular Probes) with calcein-acetoxymethylester (calcein-AM) for live cells and ethidium-1 (EthD-1) for dead cells. Calcein-AM and EthD-1 solutions were diluted to final concentrations of approximately 2 and 4 *μ*M, respectively, in the same vial of PBS solution. Samples were incubated with the reagents at room temperature for 20–40 min before confocal imaging. All stained samples were examined using a FluoView FV1000 confocal laser scanning unit with an IX81 inverted microscope (Olympus). Confocal images were processed using IMARIS software (Bitplane) for further quantitative analysis of Figs. 2 and 3. For Fig. 5, Angiotool was used for quantification on angiogenesis sprouting.^30^

## SUPPLEMENTARY MATERIAL

See the supplementary material for a detailed approach to modeling a microfluidic skin irritation test. To integrate skin layer and vasculature, we considered the cell configurations and culture conditions in the microchannel. We supplemented additional results for supporting our model.
